# On the persistence of race: Unique skulls and average tissue depths in the practice of forensic craniofacial depiction

**DOI:** 10.1177/03063127221112073

**Published:** 2022-07-23

**Authors:** Lisette Jong

**Affiliations:** University of Amsterdam, Amsterdam, The Netherlands

**Keywords:** race, face, craniofacial depiction, forensic anthropology, material-semiotics

## Abstract

The (re-)surfacing of race in forensic practices has received plenty of attention from STS scholars, especially in connection with modern forensic genetic technologies. In this article, I describe the making of facial depictions based on the skulls of unknown deceased individuals. Based on ethnographic research in the field of craniofacial identification and forensic art, I present a material-semiotic analysis of how race comes to matter in the face-making process. The analysis sheds light on how race as a translation device enables oscillation between the individual skull and population data, and allows for slippage between categories that otherwise do not neatly map on to one another. The subsuming logic of race is ingrained – in that it sits at the bases of standard choices and tools – in methods and technologies. However, the skull does not easily let itself be reduced to a racial type. Moreover, the careful efforts of practitioners to articulate the individual characteristics of each skull provide clues for how similarities and differences can be done without the effect of producing race. Such methods value the skull itself as an object of interest, rather than treat it as a vehicle for practicing race science. I argue that efforts to undo the persistence of race in forensic anthropology should focus critical attention on the socio-material configuration of methods and technologies, including data practices and reference standards.

In this article, I present a material-semiotic analysis of one way in which race comes to matter in everyday forensic practice.^1^ I focus on a practice concerned with the identification of human remains working from and with the skull, namely the making of facial depictions based on the skulls of unknown deceased individuals.^2^ Although a biological notion of race has been refuted and typological race theories have been denounced in forensic anthropology, racializing categorization practices remain integral to the process of making forensic craniofacial depictions.

In line with the proposition of M’charek and Schramm (2020), I theorize race through face-making practices, studying race as a material-semiotic object (M’charek, 2013). A material-semiotic approach understands race as a relation between various entities, of both matter and meaning, enacted in situated practices (Haraway, 1988; M’charek, 2013; Mol, 2002). Such a relational approach to race enables one to attend to the different materialities of biological race without fixing and naturalizing it (M’charek, 2013, p. 424). It puts in focus the various entities that, linked together, produce race in inherently unstable and irreducible configurations. Thus, a material-semiotic approach sensitizes the researcher to address specific articulations of race in local contexts (Balkenhol & Schramm, 2019; Van Oorschot, 2020). By ethnographically following the process of making a forensic craniofacial depiction I aim ‘to denaturalize the face as a neutral abstraction and to focus on the relations through which it *comes about*’ (M’charek & Schramm, 2020), while paying attention to when, where and how race comes to matter in the face-making process.

The prediction of facial characteristics of unknown deceased individuals presupposes a relation between the skull and the face. Gerasimov (1971), the founder of a prediction method on which present day methods are built, approached the face as ‘a simplified version of the complicated skull shape’ (p. 23). Although this assumes a direct relation between an individual skull and an individual face, in the practice of making forensic facial depictions the relation is mediated through typologies and population data. It is in such classification and translation practices that race comes to matter. Importantly, race surfaces not only in skull shape typologies that evoke 19th century racial classification systems, but also in the material organization of data and reference standards.

In this article I elaborate on three aspects of the process of making a forensic craniofacial depiction: the use of reference standards for the estimation of the ancestry of the skull, the selection and application of soft tissue depth data and the prediction of facial features. The ‘texturing’ of the depicted face (i.e. the application of facial hair, skin texture and tone, and hairstyle) allows for more artistic interpretation (Wilkinson, 2010) and is not part of my analysis here. Although interesting things can be said about what becomes of race in this final phase of the facial depiction and its circulation in the media (Nieves Delgado, 2020; Wilkinson, 2020), my focus is on the aspects of the face-making process that are notably informed by scientific research and more or less standardized methods. It is important to address how race figures in scientific practices, precisely because social constructivist understandings of race have tended to focus our attention on race as a societal matter rather than race as a matter of science (Hartigan, 2008; M’charek & Van Oorschot, 2019).

## Methods

In 2016 and 2017 I conducted ethnographic fieldwork on the disciplines of craniofacial identification and forensic facial depiction, in Europe, the United States, and Australia. I spent three months in a lab where facial depictions based on skulls are produced; I interviewed practitioners and attended workshops and conferences in the field. In the lab, I was given the opportunity to learn how to make craniofacial depictions myself. As the lab director told me: ‘The best way to learn what we’re doing is by doing it yourself.’ In this article, I draw on my own learning experience to take the reader through the process of making forensic craniofacial depictions. Being in the position of an apprentice allowed me to experience and engage with the material aspects of the process. Approaching my object of research through practice also fostered appreciation for the work being done and I am thoroughly thankful to everyone in the lab who gave me the opportunity to experience and learn.

The course of the article follows the steps of ancestry estimation, application of soft tissue depth markers and the prediction of facial features. The context of the research lab and its academic approach to the practice, thoroughly shaped my understanding of the process. The practitioners who mentored me were trained in forensic anthropology and/or anatomy and had access to academic resources. I am aware that the work environment of freelance forensic artists who are not in academic or research institutions may look quite different.

In what follows, parts of the process in which race comes to matter are highlighted and foregrounded in order to examine *how* race matters. My account of the process of craniofacial depiction does not aim to give a full or complete picture. Rather, this partial story is an effect of the guiding concern about the persistence of race in forensic anthropology. Before further explicating the process of making a craniofacial depiction, though, I will sketch some ways in which race figures in the discipline of forensic anthropology.

## Race in forensic anthropology

Race has a long and contested history in biological anthropology (Blakey, 2021; Marks, 2010; Stocking, 1982; Visweswaran, 1998). It was not until after the atrocities of WWII that, in the 1950s and 1960s, UNESCO statements on race established an understanding that in the sciences concerned with human variation, there is no biological foundation for the race concept.^3^ In these statements, race was presented as a social construct, while the term ‘ethnic groups’ was used to refer to socio-cultural differences between humans. The clinal or population model that the study of human genetics brought forward was to replace the use of the concept of race in the study of biological human differences (Montagu, 1964). This strategy for allocating the problem of race to society and removing race from science, however did and does not hold.^4^ Race was never really left behind in the sciences, including genetics.^5^

Forensic anthropology shares a history with biological anthropology and hence a complicated relation with the concept of race (Marks, 2008). Forensic anthropology is an applied version of biological anthropology concerned with the identification of unknown human remains, amongst other activities. As an applied practice, dealing with forensic casework of societal significance, forensic anthropology operates on the intersection of science and society, not as separate spheres, but rather in complex entanglements (e.g. M’charek, 2016). Hence, the bifurcation of science and society inherent to the UNESCO statements mentioned above is incongruous in an applied practice like forensic anthropology. Results of forensic anthropological analyses of human remains are communicated to the police, and sometimes the wider public is asked to help solve cases and identify unknown individuals. Thus anthropological knowledge, including statements about race and ancestry, travels and reaches publics outside the lab and academia. For forensic anthropologists, this comes with a problem of translation that echoes the science/society bifurcation: How do the categories used to classify human remains in the lab relate to the categories used in society to identify living people as part of different populations?

In forensic anthropological casework knowledge about the unknown individual is generated by situating the remains in a population. As one anthropologist puts it: ‘[F]orensic anthropology bought the roundtrip ticket, summoning the populational data back to infer the biological profile of isolated individuals’ (Cabo, 2012, p. 199). The unknown individual is categorized to belong to a population group based on a comparison of the skeletal remains with population-based reference data. In other words, to produce differences between individuals, anthropologists assume similarities within a population (see M’charek, 2000). The ‘biological profile’ includes sex,^6^ age, stature, the presence of injuries or pathologies, and ancestry. It is explicitly in ancestry assessment that the trouble with race and translation becomes a concern of practicing forensic anthropologists. According to an (in)famous 1992 article by forensic anthropologist Norman Sauer with the subtitle ‘If races don’t exist, why are forensic anthropologists so good at identifying them?’:

To be of value the race categories used by forensic anthropologists must reflect the everyday usage of the society with which they interact. In ascribing a race name to a set of skeletonized remains, the anthropologist is actually translating information about biological traits to a culturally constructed labelling system that was likely to have been applied to a missing person. (Sauer, 1992, p. 109)

Following Sauer, the anthropologist as translator re-establishes a link between the ‘culturally constructed labelling system’ and ‘biological traits’ in terms of racial categories. Smay and Armelagos (2000, p. 25) point out that such an approach, despite claiming the non-existence and invalidity of biological race, reiterates a biological foundation for the concept of race with every ancestry estimation that is performed.

Attempts to undo race in the discipline of forensic anthropology took the shape of changes in discourse and terminology. Population categories were no longer considered indicative of race but rather of, for example, biogeographical ancestry (e.g. Shriver et al., 2003) or biological affinity (e.g. Berg & Ta’ala, 2014). As Albanese and Saunders (2006) argue, changes in terminology, such as the replacement of ‘race’ with ‘ancestry’, do not challenge the underlying assumptions about human variation that hold racializing effects. The use of ancestry estimation in forensic anthropology is a recurring matter of debate in the field.^7^

Recently, the debate was stirred by Bethard and DiGangi (2020) in the *Journal of Forensic Sciences*, with a call to abandon the practice of ancestry estimation. Their letter to the editor elicited a response by Stull et al. (2021) arguing for the usefulness of ancestry estimation as part of the biological profile in forensic investigation. While they ‘accept that the race concept is far too simple for human biological variation’, Stull et al. (2021) maintain that: ‘skeletal features can be used to make predictions about probable social race groups because of their correlations to local population distributions’ (p. 417). DiGangi and Bethard (2021) responded with an article in the *American Journal of Physical Anthropology* that elaborates their argument that ancestry estimation perpetuates race science. They specifically address the use of macromorphoscopic trait lists, the observational study of characteristics of specific features of the skull, in ancestry estimation as ‘a racist harm hiding in plain sight, rarely interrogated or challenged’ (DiGangi & Bethard, 2021, p. 2), because the method rests on typological assumptions rather than thorough inquiry into the heritability of traits. In addition, they call attention to how the use of ancestry estimates in forensic investigation might hinder identification and reinforces the public’s belief in the concept of biological race, amplified by structural racism and racial bias. Invoking forensic anthropology’s responsibility to society, they emphasize that ‘reinforcing the biological race concept is not compatible with Justice’ (DiGangi & Bethard, 2021, p. 10) and maintain that the discipline’s way forward is to cease the practice of ancestry estimation.

In this article, I argue that challenging the reification of race as biological difference in forensic anthropology indeed requires more than replacing terms or shifting ideological assumptions, because race does not come about as just a matter of language. Race is better understood as a material-semiotic phenomenon that takes shape in practices (M’charek, 2013). A focus on discourse does not suffice to defy race in the discipline and in fact gives space for race to persist in methods and technologies. As such, the story of race in biological anthropology resonates with the STS trope of hybrids thriving in a bifurcated world (Latour, 1993).

## Forensic craniofacial depiction

Facial images or sculptures based on the skull of an unknown individual have a place both in archeology, for example as part of museum displays, and forensic practice. In forensic cases, craniofacial depictions can be mobilized as a last resort to assist in the process of the identification of unknown deceased individuals when other technologies of identification such as a missing persons register, DNA databank and dentistry records, did not lead to a match. The aim is for the facial depiction to resemble the face of the individual to the extent that the person can be recognized by the people who knew them in life. The resulting facial depiction can take shape as a two-dimensional drawing or computer image, or as a (photographed) three-dimensional sculpture. These images are then spread via media to gather public attention for the case and elicit recognition. The facial depiction serves as a tool in forensic investigation and does not qualify as evidence; other technologies (e.g. DNA matching) come into play to establish identification.

The methods used to produce forensic craniofacial depictions build on the premise that all skulls are unique and that these unique features of the skull form the basis for the unique facial features of every *individual* (Wilkinson, 2004). On the other hand, it builds on the notion that differences in the distribution of facial soft tissue, that is the layer of muscles, fat and skin, correspond with particular points on the skull or ‘facial landmarks’ and that soft tissue depth values for certain facial landmarks differ on average between *groups of people*, human populations.

The first sculpture based on the skull of an unknown individual was made by the German anthropologist and sculptor duo Kollman and Buchly in 1898. To produce the face of ‘the Stone-Age woman of Auvenier’ they measured the soft tissue depths of the cadavers of 25 women from the region where the remains were excavated (Kollmann & Buchly, 1898). The first time a face was sculpted on the skull of an unknown individual in a forensic context was likely by Russian anthropologist Gerasimov in the 1930s. According to his own writings, his sculptures and drawings contributed to solving many criminal cases (Gerasimov, 1971). Gerasimov’s so-called ‘anatomical method’ involves the sculpting of the facial muscles on the skull and a careful study of the relation between the shapes of the skull and the facial features. The extent to which Gerasimov relied on average facial soft tissue depth data is still a matter of debate, as these aspects of his practice are not well documented or translated (Ullrich & Stephan, 2011, 2016).

In the practice of forensic craniofacial depiction, the individual is embodied by the skull, considered to be a uniquely shaped object. But the skull does not make a face by itself. It is through population categories packaged in the biological profile, soft tissue depth data and standards for the prediction of facial features, amongst other things, that the skull can gain a face. As such, the production of a forensic facial depiction is an interplay between the individual skull and population data. It is the task of the forensic artist to make these objects of knowledge work together.

Critics of the use of facial depictions in forensic cases sometimes focus on and emphasize the role of the soft tissue depth data sets. For example, regarding the use of population-based average soft tissue depth data, physical anthropologist and anatomist George Maat warns that this method leads simply to the creation of generic faces:

Finally, the act of covering different skulls with the same mean thickness of soft tissue will make them look more alike, more average. Repeating the process would even make them indistinguishable. In fact, due to the reconstruction process, originally distinct skulls become depersonalized and equalized. Instead of being restored, they become more deprived of personal identity. (Maat, 1998, p. 252)

However, such criticisms gloss over the careful and attentive work that goes into the making of forensic craniofacial depictions and obstructs inquiry into the precise ways that race comes to matter in practice. In the process of giving a face to an unknown individual’s remains, creating space for the skull to articulate its individuality through its materiality is considered crucial for good practice. Staying close to this mode of attentiveness also leads to clues for a mode of doing differences that does not reiterate but problematizes racial classifications in the field of craniofacial research and forensic facial depiction, issues to which I return in the latter part of the article.

## Ancestry estimation

Although the skull comes with a forensic report based on the analysis of the remains in the forensic laboratory, practitioners also perform their own analysis of sex, age, and ancestry of skulls. On my first day of fieldwork in the lab I was presented with a pile of books to learn about craniofacial anatomy and analysis of the skull. Morphological approaches to ancestry estimation from the skull formed a substantial part of this literature.^8^ Although some of the (older) literature I was given to study referred to the typological classification systems in terms of different human races, in the lab, and many other places I visited, I was told that this was not correct. Instead, differences were to be understood in terms of ‘ancestry’ and the different categories referred to as ‘skull types’. Ancestry was a matter of biology while race was relegated to the science’s past.

After a short training session in sex and ancestry estimation, I was given my first practice skull to work with.^9^ While I was analyzing the 3D model of this skull, one of the researchers in the lab printed some sheets for me. These summarized the literature on sex and ancestry estimation and the prediction of facial features. He also gave me two different templates to use for the assessment. Tables 1 and 2 represent excerpts of these, illustrating the structure of the templates.

**Table 1.** Skull ancestry estimation.

Element	Assessment	Estimation
Profile		
Cranial shape		
Occipital contour		
Supra-orbital ridge		
…		
Determination:		

**Table 2.** Ancestry determination.

Feature	Caucasoid	Negroid	Mongoloid	Australoid
Profile				
Brow ridges				
Palate shape				
Incisors				
Nasal spine				
….				

The templates are supposed to assist the novice in focusing on the features of the skull considered relevant for ancestry estimation as specified in the first column of each template. The assessor describes these features in the empty boxes using sets of given terms. To distinguish between the skull types and enable comparison, the nasal bones, orbits and other features should be described in specific ways.^10^ The shape of the nasal root, for example, can be described as ‘steepled’, ‘tented’, or ‘rounded’, and the nasal aperture in terms of its width (narrow to broad). These and other terms can be found in reference tables that list the characteristics considered typical for each ancestry group. After describing the characteristics of the features, the assessor is to arrive at an estimation of the skull’s ancestry. The differences between the organization of the two templates are indicative of how different ways of organizing data and classification procedures enact the object of analysis differently. The first template invites one to first describe the features specified in the first column. It allows the assessor to pause before coming to a conclusion. The second template forces the assessor to simultaneously describe and classify. The description of the listed feature needs to be put in a box that is by the organization of the table already made to signify one of the racially classified ancestry categories. In contrast, the first template encourages the assessor to first describe the skull in terms of its features before subsuming these under any ancestry category.

The names for the skull ancestry categories, ‘Caucasoid’, ‘Negroid’, and ‘Mongoloid’ are already evocative of race. As Schramm (2020) notes, such categories ‘are not self-evident, but are relational; they are articulated through each other and in concert with particular visual conventions that are historically embedded and locally specific’ (p. 7).^11^ The names in the templates resonate with the racial classification systems introduced by 18th and 19th century European scientists who tried to capture the appearance and behavior of ‘the people of the world’ in terms of racial difference. In the resulting typologies, race was used simultaneously as a descriptive tool and an explanatory model. Phenotypical differences between humans were clustered in race categories while the categories themselves became explanations for these (and other) differences. This mode of describing and explaining differences in one act of categorization is key to such a typological approach to race. Although the biological foundation for understanding human differences in terms of racial types has been refuted, this logic persists in the practice of ancestry estimation from the skull. Specific features (e.g. different nasal bone shapes) are at the same time indicative of, and explained by the ancestry categories. Perhaps even more than the words above the columns in the tables, it is this subsuming nature of the classificatory practice of ancestry estimation that enacts race as typology. Hence race is not undone by replacing the term with ‘ancestry’ or by renaming the categories in terms of geographical locations (African, Asian, European) because the typological approach on which ancestry estimation rests is ingrained in reference materials like the tables and templates. Thus, race is reproduced as an effect of the material practice of ancestry estimation itself.

Submitted to ancestry assessment, the skull becomes a racialized object. However, not all skulls neatly fit the classification system. As any classification system, this one produces a residue of things that do not fit (Bowker & Star, 2000). Skulls in all their uniqueness and complexity are difficult objects to classify and may be rendered ‘ambiguous’. Skulls themselves may thus problematize the classification system. A practitioner explained to me in an interview that human admixture creates an endless amount of variation that makes it very difficult to assign any one individual skull to a single category. And it is precisely the variation, also within one skull, that is important to understand for making a craniofacial depiction. ‘You can have a skull that overall looks rather Caucasoid but then the mouth could be more Negroid and that makes a difference for what the face comes to look like’, he explained. To account for the variation within, this practitioner thus divides the skull into parts. He approaches the skull as a collection of different elements. Note that he does employ racial categories to describe the overall skull shape and the mouth in a typological but not mutually exclusive way.

The subsuming logic of race shapes the practice of ancestry estimation and seeps through the process of giving a face to the skull of an unknown individual, despite practitioners’ efforts to account for ambiguous skulls and highlight individually different compositions. But it is not the only logic that operates within the making of craniofacial depictions: Different classification practices can help to formulate an otherwise to the race-based logic that persists in ancestry estimation.

## Soft tissue depth data

The use of average soft tissue depth tables materialize the relation between skull ancestry and the distribution of facial soft tissue. The translation from ancestry classification to the selection and application of population-based average soft tissue depth data, carries over a racialization of difference. However, different concerns that matter in choosing soft tissue depth tables may destabilize this effect.

**Figure 1.**
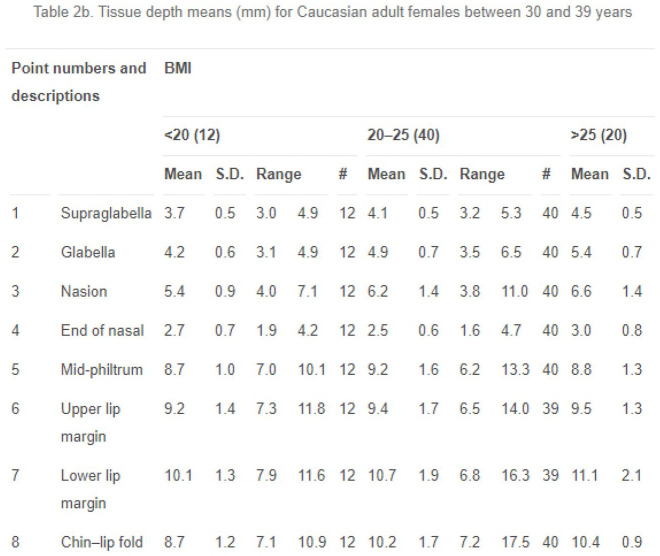
Excerpt of table in De Greef et al. (2006).

For each craniofacial depiction a set of soft tissue depth data is chosen to work with in accordance with the biological profile of the unknown individual – or, this is at least what the guidelines prescribe (e.g. Wilkinson, 2004, p. 175). Choosing a data set to work with requires a translation from the categories used to analyze skulls to the categories used to organize soft tissue depth data. ‘Caucasoid’, ‘Negroid’ and ‘Mongoloid’ are not the only categories found to refer to populations in soft tissue depth tables. More often categories of nationality, ethnicity or skin color are used to refer to the populations in soft tissue depth studies. In titles of research articles the data is, for example, presented as follows: ‘Japanese females’ (Utsuno et al., 2014) ‘Chinese-American adults in New York City’ (Chan et al., 2011), ‘black and coloured South African children’ (Briers et al., 2015), ‘adult Egyptians’ (El-Mehallawi & Soliman, 2001). Another article presents separate tables for measurements on ‘White’, ‘Black’ and ‘Hispanic children’ and ‘White’ and ‘Black adults’ in the United States (Manhein et al., 2000). Soft tissue depth data is often presented in tables similarly structured to the excerpt presented in Figure 1.

Here, ‘Caucasian’ is the first organizing principle for the presentation of the data, followed by ‘female’, then ‘30 to 39 years of age’, and then the differentiation into three BMI categories. The table articulates differences that matter, with ‘Caucasian’ as a racializing definition of the population, as the first principle for understanding soft tissue depth variation amongst humans. About the sampling procedure for the data presented in the table, the authors write: ‘Exactly 1000 volunteers were recruited on an arbitrary basis and measured using the procedure described above. After we excluded the non-Caucasians and minors, the studied population consisted of 457 males and 510 females’ (De Greef et al., 2006, p. 129). What the authors understand under the qualification ‘Caucasian’ that they use to define their population, is not mentioned in the article. Caucasian is in everyday language often used interchangeably with ‘White’. But from the anthropological literature we learn that differences in skin tone and skull shape do not neatly map onto one another (Lee et al., 2011). In addition, skull ancestry analysis does not predict skin tone, nor ethnicity or nationality. By labeling their sample ‘Caucasian’ and linking this classification with average measured tissue depths intended for use in craniofacial depiction, the authors produce a racialized population, despite anthropological reservations about the relation between skull shape and such categories.

In practice, the biological profile mediates the translation between the individual skull and the population-based average tissue depth data. But there are more things taken into account when choosing a set of soft tissue depth data to work with. The context in which the skull was found matters. For example, for a young person’s skull categorized as ‘Caucasoid’ found in an archeological site in the Netherlands, the practitioner picked a set of tissue depth data from a White British population rather than an American population. Although she said that it probably wouldn’t matter much for the reception of the final product. This was an archeological project and not a forensic case, and so recognition was not the aim. Her preference was to use data that was geographically as close as possible to the population to which the skull she was working with most likely belonged to.

The researchers in the lab where I did my fieldwork also had other concerns when choosing and applying soft tissue depth data to produce their facial depictions.^12^ For example, the measurement techniques used to collect the data make a difference for deciding how to place the markers that indicate the tissue depths on the skull. In addition, whether the individuals were measured in upright position or lying down informs how the data should be translated to the skull to create an upright facial appearance. And were the measurements taken from dead or living individuals? The aim of forensic craniofacial depiction is to produce an approximation of what the living individual looked like in life, so that the person may be recognized by relatives. The post-mortem process influences the condition and distribution of the soft tissues in relation to the skull. Hence, using soft tissue values measured on dead bodies may lead to a ‘dead’ appearance in the depiction. Therefore, data gathered from living individuals was preferable. For the practice skull I worked with, known to be derived from a Brazilian man, I was advised to use the ‘Caucasian’ soft tissue depth data produced in Belgium from living individuals, although there was a ‘Brazilian’ soft tissue depth data set available from measurements on deceased individuals. It was the initial classification of the Brazilian man’s skull shape as typically ‘Caucasoid’ that allowed me to use ‘Caucasian’ tissue depth data. Indeed, the subsuming logic of race afforded this move across categories.

Working with my practice skull and the ‘Caucasian’ tissue depth data, I cut the tissue depth marker for facial landmark ‘M2’ according to the mean value given in the table. When I glued the marker on the skull I imagined that following this soft tissue guideline would create a strangely protruding bulge on the cheek. The predicted tissue depth seemed out of proportion with the rest of the face and not fitting with the skull shape at all. I asked my mentor what to do and she replied: ‘Nobody is an average, you know!’ She then advised me to look at the range of the measured values for that landmark in the study and to cut a new marker within that range according to my best judgment for this individual skull. Handbooks on craniofacial depiction also emphasize that the unique shape of each skull should lead in the process:

Strict adherence to the exact tissue depth measurements during the craniofacial reconstruction procedure should be avoided. Experience suggests that the tissue depth data may often suggest depths that appear inappropriate for the skull, even when the data are from the correct sex, age and ethnic origin groups. In these cases the direction of the skull and the anatomy should be followed, with tissue depth measurements being used only as guides. (Wilkinson, 2004, p. 156)

Taking the materiality of the skull seriously thus means not letting the skull be reduced to an average. As Wilkinson continues:

Although the tissue depth data are very important, it must be noted that these are only mean sets of tissue thickness and, as such, cannot take into account the individuality of each skull and, therefore, each face. (Wilkinson, 2004, p. 60)

Although he would never work completely without tissue depth markers, more important is to be ‘in contact’ with the skull, one forensic artist explained to me. Being in touch with the material object sensitizes the practitioner to the intricate shapes of the individual skull. Experience, gained through working with a range of different skulls, enhances this ability to attune to shape.^13^ The practitioner, by adjusting or taking out tissue depth markers, performs a negotiation between the individual skull and the population based soft tissue depth data. Some practitioners argue that the unique shape of the skull is so powerful that its individuality will be articulated in the facial depiction, regardless of the soft tissue depth data used.^14^

Thus the relation between the individual skull and the average tissue depth data is managed through tinkering. This relation is more messy and fluid than the tenth of a millimeter precision in the tables suggests. The apparent precision in the tables enacts race as a biological difference between populations defined in terms of nationality, skin color, or ethnic group. This is substantiated by the guideline that skull type or ancestry classification informs the choice of tissue depth data. The tinkering with the tissue depth markers for an individual skull shows the limitations of average tissue depth data and its race-based logic but also its affordances. Racializing the skull facilitated the use of ‘Caucasian’ soft tissue depth data from the Belgian study in making a face for the Brazilian man’s skull. The subsuming logic of race is however challenged by the materiality of the skull when it resists reduction to an average, for example when the tissue depth marker does not quite fit. Note however, that even in such instances individuality is articulated in contrast to the population data that coproduces this misfit, and involves the careful attention of the practitioner.

## Facial feature prediction

The prediction of facial features encompasses the shaping of the eyes, nose, mouth, lips, and eyebrows of the face. This is considered a difficult endeavor because the bones of the skull hold limited clues for the shape of the soft facial features. Moreover, the bones that may provide guidance, like the teeth or the nasal spine, are not always present or fully intact. This results in uncertainty around the prediction of facial features. There is not one way to get it right, although some methods are known to lead to better approximations than others. Using different methods in triangulation or addition to one another is common practice. In this section, for brevity, I focus on methods to predict the nose; similar points could be made about the prediction methods for other facial features.

The skull cast I was practicing on was no exception. A small part of the nasal bone, where the rhinion landmark is located, seemed to be missing. The CT scans, however, showed the full nasal bone and it was from this two-dimensional rendition of the skull that I predicted most of the shape of the nose. Working on my practice skull, I combined different methods for giving shape to the nose. As advised by my mentors in the lab, I combined a series of morphological methods, described in Wilkinson’s 2004 handbook, that are based on the work of Gerasimov and his students. I also used a metric method developed by Rynn et al. (2010), colloquially referred to as ‘Rynn’s method’. These two approaches provided me with useful tools to sculpt a nose for the face I was making. In addition, juxtaposing these particular methods generated an analytical contrast to address how methods can either question or reify race-based categorization practices.

Gerasimov suggested that the ‘constitution of the subject’, as a unique assemblage of facial features, played a more important role than ‘race’ in predicting a face based on the skull (Wilkinson, 2004, p. 122). Although Gerasimov (1971) repeatedly speaks of ‘racial types’, his aim was to go beyond these generalizations to create individual faces (Ullrich & Stephan, 2016). He argues that such stereotypical generalizations do not account for the variation within the perceived groups and therefore do not provide satisfying answers in the quest to predict individual faces. Facial characteristics that seem more common in certain groups and less common in others, brought together, lead Gerasimov to an understanding of differences in terms of their underlying morphology. Regarding the nose for example he concludes:

The thickness of the soft parts at the root of the nose also does not vary according to racial type but depends directly on the degree of relief of area above the orbits and on the angle made by the projection of the nasal bones. (Gerasimov, 1971, p. 52)

**Figure 2.**
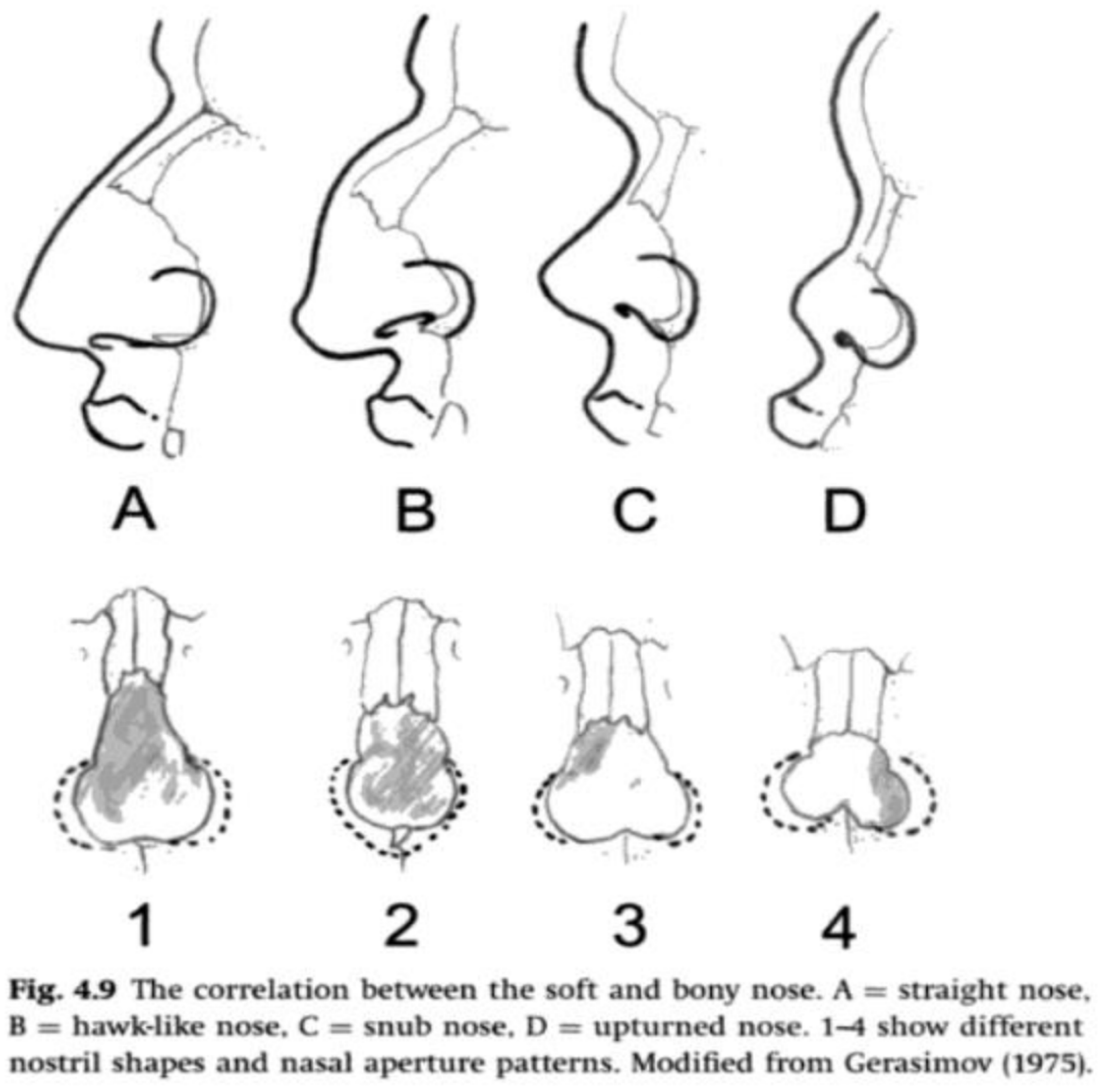
Example of a morphological prediction guideline, from Wilkinson (2004, p. 105).

The typologies for facial feature prediction created by Gerasimov and his students connect types of bone morphology to types of soft tissue facial features (Figure 2). In addition, for situating the tip of the nose the same morphological prediction procedure is applied to all skulls, the ‘two-tangent rule’: ‘A line is projected following the direction of the nasal spine. A second line, which is a tangent to the most distal portion of the nasal bones, is projected, and the intersection between the two lines should fall on the tip of the nose’ (Rynn & Wilkinson, 2006, p. 366).

**Figure 3.**
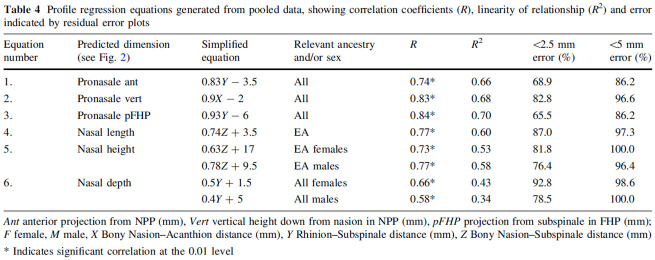
Regression equations for predicting the dimensions of the nasal profile (Rynn et al., 2010).

Rynn’s method was developed to be used in conjunction with Gerasimov’s two-tangent rule to predict the profile of the nose. The method provides six regression equations to help the practitioner situate the tip of the nose and nasal length, height and depth (Figure 3). Following these guidelines, I measured the distances X, Y, and Z on the CT scan of the skull and calculated the dimensions of the nasal profile. I drew the profile of the nose on a tracing paper over the CT scan. To give shape to the lines that connect the predicted points, I carefully studied the shapes of the nasal aperture on the skull model. Combining the knowledge about the dimensions of the nose from the metric method with morphological approaches, I sculpted a nose for the practice skull. As a novice to the face-making process, this involved a lot of trial and error, going back and forth between the sculpted nose, the skull and the predicted dimensions.

The ancestry estimation of the practice skull resulted in it being assigned to the ‘Caucasoid’ ancestry category. Therefore, next to the three equations for predicting the nasal tip, which can be used across sex and ancestry categories, I also used the equations for length and height that, following the table (Figure 3), are only applicable to ‘European ancestry’ individuals. Doing so, I thus equated ‘Caucasoid ancestry’ as it figured in ancestry estimation practices with ‘European ancestry’ as it appeared in the table, thereby racializing the relation between skull shape and nose shape.

About the use of ancestry categories in their study, Rynn et al. (2010) mention that the data pool they used was already clustered by sex and ancestry categories, based on self-classification by participants in most of the data (p. 22). About the specification of only some of the equations, the authors explain that: ‘There were too few AFR [African] and ASN [Asian] subjects (pooled *n* = 29) in this sample to produce individual rules for each ancestry group; only enough to indicate similarity or difference to the large, pooled EA [European Ancestry] group (*n* = 110)’ (Rynn et al., 2010, p. 23). The assumption that differences in nose morphology are meaningfully understood in terms of the respective ancestry groups underlies such a statement about the limitations to making ancestry group specific prediction guidelines.

Clustering data in terms of ancestry categories is common practice, but something happens when these categories are used as independent variables in prediction methods. Fixing ancestry categories as independent variables can be put in contrast with Gerasimov’s approach. His approach – staying with the skull as the object of analysis to arrive at a shape-based typology for a specific facial feature – questions everyday racial categorization practices and aims to undo race-based prediction. In the metric approach for predicting the profile of the nose on the other hand, ancestry comes to stand in for something that it is not: Ancestry is not the same thing as nose morphology, nor does a clustering of one neatly map onto the other. In other words, ancestry is made into a *proxy* for morphological difference and this puts race at the foundation of the method. Such use of ancestry categories mobilizes racialized understandings of the distribution of difference and shifts attention away from the variation within the predefined ancestry groups. Thus despite the fact that the metric approach attunes the prediction method to the dimensions of the individual skull, the use of ancestry categories as a proxy for shape at the basis of the equations has the effect of reproducing racialized understandings of sameness and difference. In addition, it encourages race-based classification practices like ancestry estimation from the skull as this becomes a precondition for deciding which equations to use.

In the process of predicting the face of an unknown individual, attuning to individuality is mediated by population categories. Race comes to matter as an effect of how these population categories are done. In ancestry estimation, working with the soft tissue depth data and some methods for facial feature prediction, ‘race as device’ (M’charek & Van Oorschot, 2019) operates as a mediator between skull and face. In moving between an individual skull and population data, race tends to subsume everything. Mitigating this tendency requires careful efforts from practitioners to articulate the individual characteristics of each skull. Haptic and experiential knowledge of shapes, developed through working with many different skulls and a thorough understanding of the way in which reference data is produced, amongst other things, enable such careful attunement in practice.

Although a race-based logic is integral to several methods and technologies employed in forensic craniofacial depiction, attending to methods based on morphological analysis showed that racialization is not necessarily inherent to the process and outcome of facial feature prediction. When prediction methods stay close to the skull, instead of resorting to race as a proxy for shape, race-based categorization tends to be destabilized rather than reified. In the following section I elaborate on what different ways of doing difference could look like in the field of craniofacial research and how these may undo race.

## Different ways of doing difference

The relevance of a race- or ethnicity-based organization of soft tissue depth data has been problematized in the field of craniofacial identification. In 2008, Stephan and Simpson (2008) published a review in which they question ‘the usefulness of the existing data subcategorizations’ for facial soft tissue depth data (p. 1257). Since differences between group averages are often small and within group variation is large, the authors argue that the uncertainty around measurements in facial soft tissue depth studies and the expected measurement error rates could in principal explain a lot of the assumed group differences. They also question the practical relevance of the often relatively small differences between population-based averages for the work of making craniofacial depictions. On the ‘racial organization’ of soft tissue depth values they write:

Overall, these findings suggest that ‘race’ effects on soft tissue depth data are not strong since studies display broad but similar soft tissue depth ranges and central tendencies irrespective of ‘race’. Furthermore, any race differences that do exist are likely overpowered by differences between measurement methods. (Stephan & Simpson, 2008, p. 1264)

Thus, following the authors, due to statistical uncertainty and measurement effects, group differences found in facial soft tissue depth studies cannot be attributed to variables of race or sex, whether or not these produce ‘real’ effects on facial soft tissue depth distribution. This then undermines the assumption that using race- or sex-specific soft tissue depth data leads to more accurate craniofacial depictions. In their study, Stephan and Simpson find differences between broad age categories to be significant and suggest working with two pooled data sets, one for children and one for adults, that are not differentiated any further. These data sets pool together the weighted means from all soft tissue depth studies included in their research. The authors have set up an online database and encourage researchers to add new measurements to further improve the pooled data sets and enable different kinds of analyses to be conducted.

While Stephan and Simpson’s critical study of methods and statistical inferences in soft tissue depth research is appreciated in the field, their solution of universally applicable data sets is not immediately accepted. One forensic artist commented that the ‘one size fits all’ approach to soft tissue depth data does not take morphological differences into account while ‘morphology overrides everything’. The suggestion is that soft tissue depth data subcategorizations are a meaningful way to attend to differences in skull and facial shape. Another practitioner was mostly critical of the pooling of data from dead and living individuals, showing me an article in which facial depictions based on cadaver data came out looking like dead faces. The pooled data set erases differences that matter, according to these practitioners, and are precisely therefore not universally applicable.^15^

A universal data set is not the only alternative to data practices that employ race and sex categories as proxies for morphological differences. When I asked an expert in the field about the possibilities for different ways of organizing tissue depth data, she mentioned a study by Utsuno et al. (2010, 2014). The authors propose a differentiation of soft tissue depth measurements based on occlusion types, that is the relation between the upper and lower teeth when the jaw is closed. As all occlusion types are found in all ancestry groups, predicting soft tissue depths based on occlusion would lead to more accurate facial depictions than using ancestry as a proxy for shape. Such shape-based organization of tissue depth data could be the future of craniofacial research, the expert suggested, although she considered it unlikely that this shift would happen rapidly.

Race has been a driving force in the collection of anthropometric data since the early days of physical anthropology. The proliferation of soft tissue depth data sets for different populations is entangled with that history. The quest for more specific soft tissue data, in order to produce more accurate craniofacial depictions, took form as a quest for more data on more groups differentiated in terms of ethnicity, nationality or race. Categories of ancestry, sex, and age have been enrolled in data practices as proxies for shape and serve to make findings compatible and comparable. As such, the historical relevance of race has sedimented in scientific practice and persists in present day methods of data collection and organization. A shift toward a shape-based organization of soft tissue depth data is complicated by this dependence on past data practices in the discipline. In addition, reassessment of older data sets that are still in use today would be a huge if not impossible undertaking as relevant skull and facial shape characteristics have most likely not been recorded or have been erased and subsumed by race-based categorization practices.

However, there are efforts to move the field of craniofacial research in the direction of a shape-based organization of tissue depth data. At the 2017 conference of the International Association for Craniofacial Identification, Louisa Baillie gave a keynote lecture titled: ‘Can craniofacial soft tissue prediction be improved by considering distinct facial bone morphology rather than ancestry?’ The subsuming effect of categorizing an entire skull to one ancestry group can be avoided, but requires a change in method, Baillie argues. The proposed alternative involves a clustering of soft tissue depth data based on shape differences and similarities at the level of parts or ‘regions’ of the skull. This ‘regional bone morphology approach’ also allows one to account for the variation within one individual skull as the entire skull is not reduced to any one population group. It thus facilitates staying close to the skull as the object of analysis.

Clustering data in terms of shape categories, the regional bone morphology approach provides an alternative to the race-based categorization of soft tissue depth data. This does not necessarily mean that predefined population categories did not play a role at all in Baillie’s work. The research materials were initially categorized and compared as comprising data from Chinese and European New-Zealand women respectively (Baillie et al., 2015).^16^ But, importantly, this is not the level at which conclusions about differences and similarities are drawn as we saw happening in the tissue depth table in Figure 1 and Rynn’s equations in Figure 3. Rather, such everyday perceptions of difference are taken as an invitation for research and exploration, thereby shifting ‘the logic of difference’ away from racial categories as explanatory ends in themselves (Shim et al., 2014). A shape-based regional bone morphology approach allows for differences that matter to appear instead of being obscured by the subsuming logic of race or the application of universal standards. It holds a promise to both improve prediction accuracy, as Baillie et al. (2017) argue, and to methodologically undo race.

## Conclusion

Though discursively put aside, race is not left behind in forensic anthropology, as it is ingrained in everyday methods and technologies. As Fullwiley (2015) notes: ‘when technologies are born of race sorting logics, then the resultant race problems and their proposed solutions contain the same disturbing seed elements’ (p. 37). To effectively address the persistence of race in forensic anthropology, critical attention should be paid to the socio-material configurations of methods, including data practices, reference standards and technologies in forensic craniofacial identification practices. This is relevant to not only forensic craniofacial depiction practices, or even the field of forensic and biological anthropology. Similarly, a recent article in *The Lancet* about race-based medicine, dissects how the use of race as independent variable in biomedical and epidemiological research translates into clinical medicine and perpetuates inequitable care (Cerdeña et al., 2020). Race either implicitly or explicitly continues to shape and be shaped by scientific practices in many different domains. Careful attention to how race is being done in everyday knowledge production practices could shed light on ways to undo it in each of these instances. Opening up scientific practice for better, and likely more accurate, methods that do not resort to the use of racial categories as proxies is important. This is not least because reifications of biological understandings of race in scientific knowledge travel through society, for example as applied in forensic casework and clinical medicine, with implications for public understandings of sameness and difference and the consequence of sorting racist effects.

Race comes to matter in different aspects of the practice of making forensic craniofacial depictions. Closely following the process of face-making, I foregrounded both the affordances and the limitations of race-based categorization in everyday work practice. As a translation device, race enables oscillation between the unknown individual and population data. The logic of race allows for slippage between categories that do not neatly map on to one another. The skull, however, does not let itself be easily reduced to a racial type. It demands to be taken seriously in itself, most notably when standards fail to account for its intricate shapes. In this regard, practitioners take the response-ability to articulate the morphology of the individual skull. Tinkering with average data and attending to individual facial asymmetries are considered to be crucial steps for increasing the chance at recognition by someone who knew the unidentified person in life.

Predicting what the face of an unknown individual may have looked like when they were alive cannot be done without making use of population-based reference data. However, morphological methods of ancestry estimation, and by extension the data infrastructure on which most facial soft tissue depth studies rely, rest on practices that historically marked physical anthropology as a racial science. The skull as an object of analysis has played an important role in sorting people into types based on assumed racial differences. The scientific study of differences in skull shape and size was mobilized to provide a biological foundation for race and racial hierarchies. Present day forensic anthropological practices are motivated by the need to identify bodies in support of investigative processes. Although the discipline of forensic anthropology has left racial hierarchies behind, typological assumptions about difference are in the foundations of methods and technologies that form the basis of current work practice. This makes the discipline complicit in reproducing, normalizing and spreading a biological understanding of race (DiGangi & Bethard, 2021). Concerns about accuracy, and about the comparability and compatibility of data over time and place, add to the robustness of data practices and the difficulty to leave race-based methods behind.

Alternative approaches gives us hints at what questioning assumptions about difference could look like in facial soft tissue depth studies. A shape-based method that takes the materiality of the skull seriously has the potential to emancipate the skull from a long history of being subsumed by the principles of typological reduction. To undo race, forensic anthropology should foster data practices that value the skull itself as an object of interest rather than reduce the skull to a vehicle for practicing race science. Importantly, although alternative approaches are promising, they do not in and of themselves let forensic anthropology move beyond race. As Fujimura and Rajagopalan (2011) and Shim et al. (2014) point out in the context of research into genetic markers for complex diseases, racial categories may still shape understandings of sameness and difference in the selection and definition of study populations, as well as slip back in when results travel outside of the laboratory (see also M’charek et al., 2020). This means that, even if the practice of ancestry estimation were abolished, the need remains to be critical of where, when and how race may emerge as biology in the entanglements of forensic anthropology with science and society.

## Notes

1. The (re-)surfacing of race in forensic practices has received plenty of attention from STS scholars, especially in connection with modern forensic genetic technologies such as DNA phenotyping, familial searching and forensic DNA databases (e.g. Cole & Lynch, 2006; Duster, 2006; Hopman & M’charek, 2020; M’charek, 2000, 2020; M’charek et al., 2020; Ossorio, 2006; Queirós, 2019; Sankar, 2010; Skinner, 2020).

2. Making facial depictions based on unidentified skulls is a practice also known as ‘craniofacial reconstruction’ (Taylor, 2000; Wilkinson, 2004) or ‘facial approximation’ (Hayes et al., 2005; Stephan, 2015). In this article, I use the term (forensic) craniofacial depiction (e.g. Roughley & Wilkinson, 2019) to encompass different 2D and 3D presentations of the final face.

3. This was however a process. The statements from 1950 and 1951 were not about undoing race as biology, but rather about separating the scientific facts about race from (Nazi) ideologies about race (Reardon, 2004). Thereby, the statements cultivated a space for race in science and maintained physical anthropologists as the experts on racial classification. It was only in the 1967 revisions that the use of racial classification for scientific purposes was explicitly problematized.

4. By making ‘population’ the privileged object of knowledge, the UNESCO statements importantly challenged (although not defeated) typological approaches to race and opened up the question of what was then to count as a population (Haraway, 1989, p. 202). However, this openness also gave way for translations of the population concept, for example in terms of ‘social group’, that reaffirmed racialized understanding of sameness and difference in biological anthropology’s post-UNESCO anti-racist efforts (Haraway, 1989).

5. Many STS scholars, among others, have attended to the persistence of race in genetics (e.g. Duster, 2005, 2015; El-Haj, 2007; Fujimura & Rajagopalan, 2011; Fullwiley, 2007; Lipphardt, 2014; Montoya, 2007; M’charek, 2013, 2014; Reardon, 2004; Selcer, 2012)

6. While this article is focused on categories of ancestry and race, the use of sex as a binary category in the biological profile can similarly be problematized. See, for example, the work of Pamela Geller (2008) in the field of bioarcheology. How this affects the identification of missing and unidentified bodies of trans and non-binary individuals in forensic cases, is addressed by the Trans Doe Task Force (https://transdoetaskforce.org/). Recent attempts to ‘break the binary’ in forensic anthropology focus for example on how understanding the traces left on the skull by facial feminization surgery, can assist in the identification of transgender women (Schall et al., 2020).

7. For more elaborate reviews of the debate about the concept of race and ancestry estimation in forensic anthropology see Smay and Armelagos (2000), Ousley et al. (2018), Cunha and Ubelaker (2020). Ross and Pilloud (2021) specifically cover the debate from the 1990s onwards.

8. Methods for ancestry and sex estimation are usually differentiated between morphological and craniometric approaches. Craniometric approaches can be assisted by software like FORDISC (Jantz & Ousley, 2005). In the lab where I did my fieldwork, and by other practitioners I spoke to, a morphological analysis for ancestry estimation was considered most useful for craniofacial depiction, therefore this is what I focus on here.

9. The ‘practice skulls’ I worked with were 3D models of skulls based on CT data of the skulls of living human beings who consented to their data being used for teaching and research purposes.

10. During my fieldwork period I was also corrected when I described other qualities of features or used different terms in this part of the process: ‘This is not relevant for ancestry estimation.’

11. While these categories that are a product of European racial science are used across the world, they also are translated or adapted to fit locally relevant classification systems. Nieves Delgado (2020) for example discusses the use of the notion of *mestizo* in ancestry estimation of skulls in Mexico.

12. Situated at a university, the researchers in the lab had access to and engaged with academic literature. They were up to date on publications on soft tissue depth data. This was not the case for all the forensic artists I encountered in the course of my research. Practitioners working at the police or as a freelancer, did not as easily have access to the academic literature and were not as aware of the different tissue depth tables available. Some told me that they just tinkered with one or two different tables from a handbook they owned, for example the Rhine and Campbell (1980) and Rhine and Moore (1982) tables printed in Taylor’s (2000) handbook *Forensic Art & Illustration*.

13. In forthcoming work I elaborate on haptic knowing and address how practitioners also employ their own bodies as reference material in the field of craniofacial identification.

14. For example, in an experiment, Wilkinson (2004) had six faces sculpted on the same skull using soft tissue depth values from six different study populations. All six facial depictions had reasonable recognition ratings in the subsequent face pool test, suggesting that the use of different soft tissue depth data for the same skull does not make a big difference for recognition purposes. As she concludes: ‘The tissue depth data clearly affect the facial appearance, but the skull itself was shown to be the most important factor when producing a facial reconstruction. The correct set of tissue depth will produce the most accurate likeness, but erroneous tissue depth data will not affect the face to the extent that it cannot be recognized by a relative or a close friend’ (Wilkinson, 2004, p. 156). Note that the goal of accuracy here still gives value to working with population-based soft tissue depth data sets.

15. Stephan (2014) argues that the ‘law of large numbers’ makes up for these differences and errors. In practice, however, knowledge about where the data comes from, what measurement techniques have been used and at what points exactly measurements were taken, is taken into account when giving a face to a skull. For example, when I asked about a tissue depth value in the cheek area that seemed rather low, it was explained to me how this made sense for this particular landmark in this particular data set. It was likely that, while taking the measures, more pressure was put on the skin with the transducer because the right angle between the facial and bony landmarks is hard to make, resulting in a lower measured tissue depth value. Such knowledge is obscured by the tallied table.

16. Baillie et al. (2015) published a study in which they questioned ‘ancestry and BMI influences on facial soft tissue depths’. The authors compared the soft tissue depth values of two groups of women living in New Zealand, one group defined as having European and the other Chinese ancestry. When controlling for BMI, they found an average tissue depth difference between the groups for two facial landmarks in the area of the cheeks. There was no significant difference between the group averages for the other landmarks. Baillie referred to this data set in her keynote at the IACI conference.
